# Meaningful Interactional Diversity, Professional Development, and Service Intent in White Medical Students

**DOI:** 10.1001/jamanetworkopen.2025.60266

**Published:** 2026-02-20

**Authors:** Shruthi Venkataraman, Mytien Nguyen, Alexandra M. Hajduk, Adeola Ayedun, Will Roberts, Bassel Shanab, Allison Aviles, Nhu Y. Doan, Meghan O’Connell, Soo-Min Shin, Gbenga Ogedegbe, David Henderson, Somnath Saha, Jeph Herrin, Tonya Fancher, Sarwat I. Chaudhry, Dowin Boatright

**Affiliations:** 1 Department of Emergency Medicine, New York University Grossman School of Medicine, New York; 2 Department of Immunobiology, Yale School of Medicine, New Haven, Connecticut; 3 Section of Geriatrics, Department of Internal Medicine, Yale School of Medicine, New Haven, Connecticut; 4 National Clinical Scholars Program, Yale School of Medicine, New Haven, Connecticut; 5 Yale School of Medicine, New Haven, Connecticut; 6 Section of General Internal Medicine, Department of Internal Medicine, Yale School of Medicine, New Haven, Connecticut; 7 Division of General Internal Medicine, School of Medicine, University of California, Davis; 8 Institute for Excellence in Health Equity, NYU Grossman School of Medicine, New York, New York; 9 Department of Family Medicine, University of Connecticut School of Medicine, Farmington; 10 Division of General Internal Medicine, Johns Hopkins University, Baltimore, Maryland; 11 Section of Cardiovascular Medicine, Department of Internal Medicine, Yale School of Medicine, New Haven, Connecticut

## Abstract

**Importance:**

At a time when diversity in higher education is increasingly perceived as beneficial only to minority students and disadvantaging to majority-group students, it is crucial to investigate its benefits broadly.

**Objective:**

To assess whether meaningful interactional diversity (ie, cross-cultural engagement that influences knowledge or opinions) is associated with key educational outcomes among White medical students.

**Design, Setting, and Participants:**

This cross-sectional study included self-identified White-only medical students who matriculated to a US allopathic medical school between 2013 and 2022, graduated between 2015 and 2024, and responded to the Association of American Medical Colleges Graduation Questionnaire (GQ). GQ responses were anonymous. Data were analyzed from September 2024 to April 2025.

**Exposure:**

Meaningful interactional diversity, defined by agreement on a 5-point Likert scale with the GQ item, “My knowledge or opinion was influenced or changed by becoming more aware of the perspectives of individuals from different backgrounds.”

**Main Outcomes and Measures:**

Six self-reported binary outcomes derived from the GQ were assessed: personal and professional development fostered by medical schools, ability to care for and work with people from different backgrounds, and intention to work in an underserved area and with an underserved population regardless of practice location.

**Results:**

Among 80 542 students (38 589 [47.91%] female) from 155 medical schools, 495 (0.61%) strongly disagreed, 1416 (1.76%) disagreed, 7387 (9.17%) were neutral, 35 155 (43.65%) agreed, and 36 089 (44.81%) strongly agreed with meaningful interactional diversity. Across increasing agreement with meaningful interactional diversity, higher likelihoods of personal and professional development and care and teamwork competence were observed, whereas associations with service intentions were confined to strong agreement. Compared with strong disagreement, strong agreement was associated with greater personal (adjusted relative risk [aRR], 2.37; 95% CI, 2.05-2.74) and professional (aRR, 1.59; 95% CI, 1.42-1.69) development, competence to care for (aRR, 1.23; 95% CI, 1.17-1.29) and work with (aRR, 10.69; 95% CI, 7.84-14.57) people from different backgrounds, and plans to work in underserved areas (aRR, 1.31; 95% CI, 1.08-1.58) or with underserved populations regardless of practice location (aRR, 1.59; 95% CI, 1.30-1.93).

**Conclusions and Relevance:**

In this cross-sectional study of White medical students, meaningful interactional diversity was associated with graded improvements in core developmental outcomes and threshold-level associations with service intentions.

## Key Points

**Question:**

Is self-reported meaningful interactional diversity (defined as cross-cultural engagement influencing knowledge or opinions) in medical school associated with perceived development, professional competence, and service intentions among White students?

**Findings:**

In this cross-sectional study of 80 542 White students from 155 US medical schools, meaningful interactional diversity showed graded associations with personal and professional development as well as competence to care for and work with people from different backgrounds, whereas associations with intentions to serve underserved communities occurred only with strong endorsement of meaningful interactional diversity.

**Meaning:**

The findings of this study suggest that meaningful interactional diversity may positively influence key educational outcomes and service intentions among White US medical students.

## Teaser

This cross-sectional study evaluates whether White medical students’ experience of meaningful interactional diversity—the experience of changing one’s knowledge or opinions as a result of encountering the perspectives of individuals from different backgrounds—is associated with personal and professional competencies as well as plans to work with underserved populations.

## Introduction

Diversity in medical education is often evaluated through the lens of representation.^1,2^ Yet, it may be equally important to assess the depth of student engagement with peers from different backgrounds and to evaluate the broader educational benefits this interactional diversity may confer, including for majority-group students. While structural diversity (ie, representation) is a necessary precondition for cross-cultural exchange,^3,4^ *meaningful interactional diversity*, or interpersonal engagement between members of diverse groups in both formal and informal contexts that can influence knowledge or opinion, has been linked to cognitive, intellectual, social, civic, and attitudinal developmental gains in higher education.^4,5,6,7,8,9,10^

Scientific research on the benefits of meaningful interactional diversity in medicine has typically framed majority-group students, particularly White students, as beneficiaries of prejudice reduction, empathy, or moral development (eg, becoming less biased or more tolerant) and underrepresented groups as the indirect recipients of these gains.^5,11,12^ It is crucial to comprehensively investigate benefits of meaningful interactional diversity in medical education for majority-group members with historical privilege in an era when diversity is increasingly perceived by majority-group members as a zero-sum game—where equity initiatives are framed as redistributive losses rather than collective gains and the benefits of inclusion are presumed to accrue solely to minoritized groups.^13,14,15,16^

To address this evidence gap, this study builds on work by Saha and colleagues,^5^ who examined associations between meaningful interactional diversity, as experienced by White medical students, and competence to care for individuals from different racial and ethnic backgrounds and intentions to practice in underserved areas using 2003-2004 Association of American Medical Colleges (AAMC) data. The study by Saha et al^5^ has since catalyzed research on the roles of structural and meaningful interactional diversity in shaping educational experiences and learner development.^17,18^ Using a contemporary dataset, we examine whether associations with intentions to practice in underserved areas persist and extend this work by evaluating personal and professional development—now recognized by the AAMC as foundational medical training competencies^19,20^—along with intentions to care for underserved populations regardless of practice location, teamworking competence, and a broadened measure of care competence. Collectively, these outcomes may be important for fostering effective physicians and strengthening clinical care and public health.^5^ Prior work suggests these outcomes may relate to trainee well-being^21,22^ and workforce shortages in underserved communities^23^ and may help address the disproportionate reliance on minoritized clinicians to address health care disparities.^24,25^ This study responds to national imperatives to advance diversity science in the health care workforce,^26^ investigating how diverse learning environments can enhance educational quality and workforce preparedness.

## Methods

### Study Population

Data from the American Medical College Application Service, Student Records System, Liaison Committee on Medical Education (LCME) Part I-A Annual Financial Questionnaire among US medical schools with full LCME accreditation as of fiscal year 2017 to 2018, and Graduation Questionnaire (GQ) responses were provided by the AAMC for 80 571 self-reported White-only (ie, not multiracial) medical students who matriculated between 2013 and 2022 and graduated between 2015 and 2024. GQ responses were anonymous. Of these, 29 individuals missing data on sex (0.04%) were removed, yielding a final cohort of 80 542 students. US allopathic medical students who chose to participate in these AAMC-administered national surveys after receiving detailed information provided implied informed consent. This secondary analysis of fully deidentified data was exempt from review and informed consent per criteria from the New York University Langone Health institutional review board. The report follows the Strengthening the Reporting of Observational Studies in Epidemiology (STROBE) reporting guideline for cross-sectional studies.

### Conceptual Model

**Figure 1.**
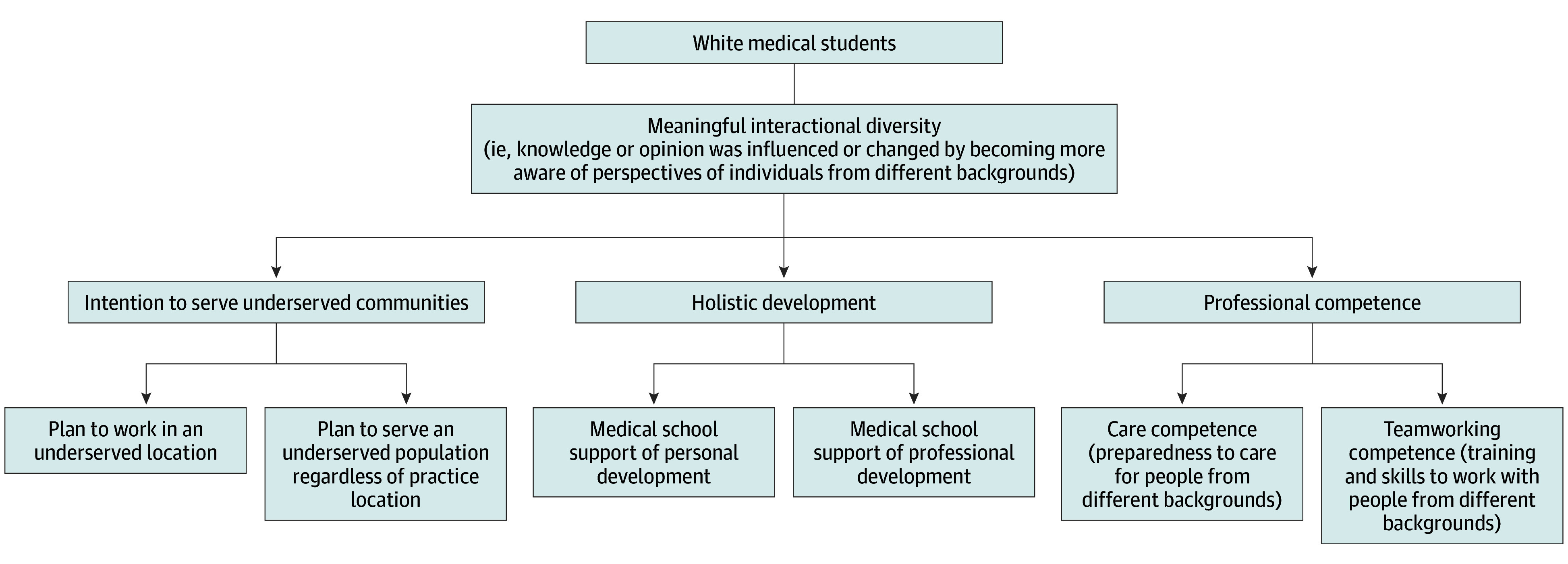
Conceptual Model

Our conceptual model (Figure 1) was based on the framework by Gurin et al,^7^ which posits interactional diversity as a mechanism through which structural diversity yields educational benefits. We build on this model by examining a broad set of outcomes for majority-group students, beyond commonly studied attitudinal or bias-reduction outcomes, to include competencies central to physician training.

### Variables

#### Sociodemographic and School-Level Characteristics

Students self-reported their sex as male or female. Students self-reported their race and ethnicity as corresponding to any or all the following groups: African American or Black; American Indian or Alaska Native; Asian; Hawaiian Native or Pacific Islander; Hispanic, Latino, or of Spanish Origin; White; other, and unknown. Only students reporting White race exclusively were included in this study. Students who received either a Pell grant or state or federal means-tested financial assistance for low-income families (eg, Supplemental Nutrition Assistance Program) were categorized as being from low-income backgrounds. Sexual orientation was categorized as lesbian, gay, or bisexual (LGB) and heterosexual. Medical school ownership was classified as public or private, and the school region as Central, Northeast, Southern, or Western.

#### Exposure Variable

Meaningful interactional diversity was assessed using the GQ item, “Based on your experiences, indicate whether you agree or disagree with the following statements: My knowledge or opinion was influenced or changed by becoming more aware of the perspectives of individuals from different backgrounds.” Responses were recorded on a 5-point Likert scale from strongly disagree to strongly agree. In this context, “different backgrounds” refers broadly to social, cultural, and identity-based differences (eg, socioeconomic status, disability, language, gender, race and ethnicity, and so on). This operationalization aligns with established research in higher education, including medical education, which has used self-reported measures of cross-cultural engagement to examine associations with cognitive, affective, and civic development.^5,7,27,28,29^

#### Educational Outcomes

Personal development was measured by the GQ item, “My medical school has done a good job of fostering and nurturing my development as a person,” and professional development was measured by the GQ item, “My medical school has done a good job of fostering and nurturing my development as a physician.” Responses were recorded on a 5-point Likert scale from strongly disagree to strongly agree. Variables were dichotomized such that only participants who expressed agreement (ie, agree or strongly agree) were considered as reporting that their medical school nurtured their development. These measures are grounded in precedent, having been similarly operationalized in prior studies.^20,30,31,32^

Care competence was measured by the GQ item, “I believe I am adequately prepared to care for patients from different backgrounds.” Teamworking competence was measured by the GQ item, “The diversity within my medical school class enhanced my training and skills to work with individuals from different backgrounds.” Both items used a 5-point Likert scale ranging from strongly disagree to strongly agree to record responses. Both variables were dichotomized to facilitate interpretability such that only those who selected agree or strongly agree were categorized as adequately prepared. We intentionally omitted the term “cultural” from these variable labels to avoid reinforcing the notion that competence in working across difference applies only to certain encounters. Because culture encompasses intersecting domains such as race, ethnicity, disability, language, gender, and more, and because cultural differences likely exist between any 2 individuals, we frame these skills as fundamental to all clinical and team interactions.^33,34^

Intention to work in an underserved area was assessed by the GQ question, “Do you plan to work primarily in an underserved area?” Intention to care for underserved populations was assessed by the question, “Regardless of location, do you plan to care primarily for an underserved population?” For both items, responses were recorded as yes, no, or undecided. For analysis, no and undecided were grouped together, while yes was treated as indicating a clear intention.

### Statistical Analysis

Data analyses were performed from September 4, 2024, to April 30, 2025, using Stata version 16.1 (StataCorp). First, student characteristics were summarized, and *P* values from *F* tests based on analysis of variance were reported. We used modified Poisson regression with robust SEs clustered at the school level to estimate the association between meaningful interactional diversity (exposure) and the 6 common, binary educational outcomes: personal development, professional development, care competence, teamworking competence, plans to work in underserved areas, and plans to care for underserved populations. This modeling approach can be used to estimate relative risks for common binary outcomes.^35,36^ The exposure variable was modeled categorically rather than ordinally in regression analyses to more accurately reflect qualitatively distinct levels of engagement with peers from diverse backgrounds, thereby avoiding potentially invalid assumptions of linearity or monotonicity. Adjacent-level contrasts of the meaningful interactional diversity exposure (eg, strongly agree vs agree, agree vs neutral, and so on) were conducted across all 6 outcomes. Models were adjusted for sex, low-income status, sexual orientation, and school ownership and region, which are characteristics known to influence career intentions, exposure to diversity, and educational experiences among US medical students.^5,37,38,39,40^ All models were also adjusted for matriculation year to account for cohort effects. Statistical significance was set at a 2-sided *P* < .05. Multicollinearity diagnostics using variance inflation factors (VIFs) revealed no concerns (VIFs <5). To contextualize school-level differences, we conducted a secondary analysis comparing structural diversity characteristics—specifically, the proportions of students underrepresented by race and ethnicity, who belonged to minoritized racial and ethnic groups, who were low-income, and identifying as LGB—between schools above and below the median for White student meaningful interactional diversity using independent sample *t* tests. Missing data were handled via multiple imputation using fully conditional specification, with 20 datasets generated. The imputation model included all sociodemographic, school-level, exposure, and outcome variables. Two separate sensitivity analyses, one using complete cases and another restricted to the 2 most recent GQ cohorts were performed. Imputed models are presented here, in accordance with best practices for multiple imputation analysis and reporting.^41,42^

## Results

### Participant and School Characteristics

For details on variable missingness, see eTables 1 and 2 in Supplement 1. A total of 50 884 of 80 542 White students (63.18%) answered all of the AAMC questionnaire items included in the study (complete cases), and historically, the overall response rate of the GQ has centered around 80%.^43^ Among participants in the imputed dataset (N = 80 542), 38 589 (47.91%) identified as female, 22 195 (27.56%) as low-income, and 7741 (9.61%) as LGB. Students were regionally distributed across the Central (23 154 [28.75%]), Northeast (21 549 [26.75%]), Southern (27 981 [34.74%]), and Western (7858 [9.76%]) US, with 52 955 (65.75%) attending publicly owned medical schools. Overall, 35 155 (43.65%) agreed and 36 089 (44.81%) strongly agreed with the GQ item assessing meaningful interactional diversity (eTable 3 in Supplement 1).

The proportion of students reporting strong agreement with meaningful interactional diversity (ie, that one’s knowledge or opinions were influenced or changed by exposure to the perspectives of individuals from different backgrounds) varied across demographic and institutional groups. Among female students, 18 887 (48.94%) strongly agreed, compared with 17 202 male students (41.00%). Among students identifying as LGB, 4034 (52.11%) strongly agreed vs 32 055 heterosexual students (44.03%). Among low-income students, 9479 (42.71%) strongly agreed, compared with 26 610 non–low-income students (45.61%). By school characteristics, 22 723 students (42.91%) at public institutions and 13 366 (48.45%) at private institutions strongly agreed. Regionally, strong agreement ranged from 9801 (42.33%) in the Central region to 10 186 (47.27%) in the Northeast, with 12 386 (44.26%) in the South and 3716 (47.29%) in the West (Table). These patterns were consistent in complete cases (eTable 4 in Supplement 1).

**Table.** Characteristics of White Medical Students and Their Schools by Meaningful Interactional Diversity (Postimputation Estimates)

Characteristic	Participants by level of agreement with meaningful interactional diversity survey item, No. (%) (N = 80 542)					*P* value
	Strongly disagree (n = 495)	Disagree (n = 1416)	Neutral (n = 7387)	Agree (n = 35 155)	Strongly agree (n = 36 089)	
Sex						
Female	116 (0.30)	427 (1.11)	2681 (6.95)	16 478 (42.70)	18 887 (48.94)	<.001
Male	379 (0.90)	989 (2.36)	4706 (11.22)	18 677 (44.52)	17 202 (41.00)	
Low-income						
Yes	143 (0.64)	461 (2.08)	2280 (10.27)	9832 (44.30)	9479 (42.71)	<.001
No	352 (0.60)	955 (1.64)	5107 (8.75)	25 323 (43.40)	26 610 (45.61)	
Sexual orientation						
Lesbian, gay, or bisexual	37 (0.48)	99 (1.28)	523 (6.76)	3048 (39.37)	4034 (52.11)	<.001
Heterosexual	458 (0.63)	1317 (1.81)	6864 (9.43)	32 107 (44.10)	32 055 (44.03)	
School region						
Central	138 (0.59)	470 (2.03)	2155 (9.31)	10 590 (45.74)	9801 (42.33)	.006
Northeast	121 (0.56)	323 (1.50)	1886 (8.75)	9033 (41.92)	10 186 (47.27)	
Southern	195 (0.70)	500 (1.79)	2642 (9.44)	12 258 (43.81)	12 386 (44.26)	
Western	41 (0.52)	123 (1.57)	704 (8.96)	3274 (41.66)	3716 (47.29)	
School ownership						
Public	335 (0.63)	990 (1.87)	5155 (9.74)	23 752 (44.85)	22 723 (42.91)	<.001
Private	160 (0.58)	426 (1.54)	2232 (8.09)	11 403 (41.34)	13 366 (48.45)	

Schools with ratings above the median for meaningful interactional diversity, compared with those below the median, had higher proportions of students from minoritized racial and ethnic groups (48.25% vs 40.12%; *P* = .004) and LGB students (8.55% vs 6.42%; *P* < .001) but lower proportions of low-income students (28.89% vs 32.19%; *P* = .04). (eTable 5 in Supplement 1).

### Associations With Personal and Professional Development

**Figure 2.**
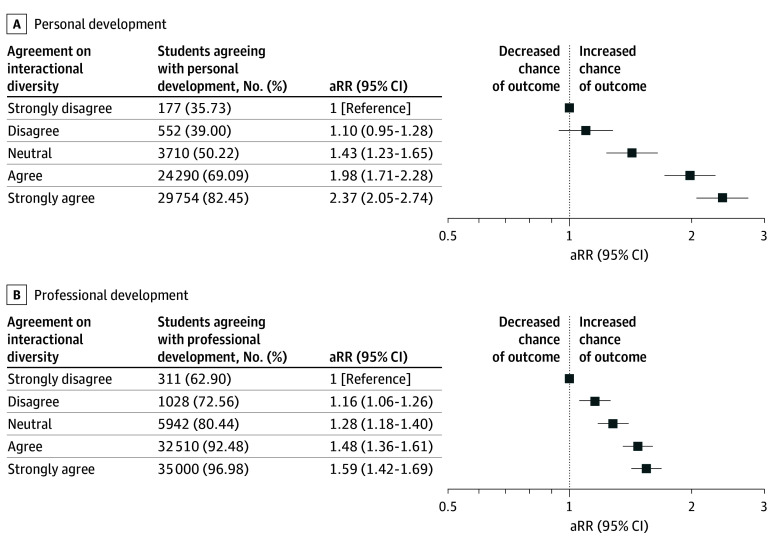
Personal and Professional Development Among White Medical Students by Meaningful Interactional Diversity Adjusted relative risks (aRRs) and 95% CIs for students’ ratings of their medical schools’ support for personal development (A) and professional development (B), stratified by agreement with the meaningful interactional diversity item. Agreement reflects the extent to which students reported that their knowledge or opinions were influenced or changed by the perspectives of individuals from different backgrounds. Models adjust for sex, low-income status, sexual orientation, school ownership, region, and matriculation year. The reference group is students who strongly disagreed.

Most students reported that their medical school fostered their personal (58 483 [72.61%]) and professional (74 791 [92.86%]) development. Higher levels of agreement on meaningful interactional diversity were positively associated with both outcomes. For personal development, adjusted relative risks (aRRs) increased from 1.43 (95% CI, 1.23-1.65) among those neutral to 2.37 (95% CI, 2.05-2.74) among those strongly agreeing, compared with those strongly disagreeing. Professional development followed a similar trend, with aRRs ranging from 1.28 (95% CI, 1.18-1.40) among those neutral to 1.59 (95% CI, 1.42-1.69) among those strongly agreeing, compared with those strongly disagreeing (Figure 2).

### Associations With Competence to Care for and Work With People From Different Backgrounds

**Figure 3.**
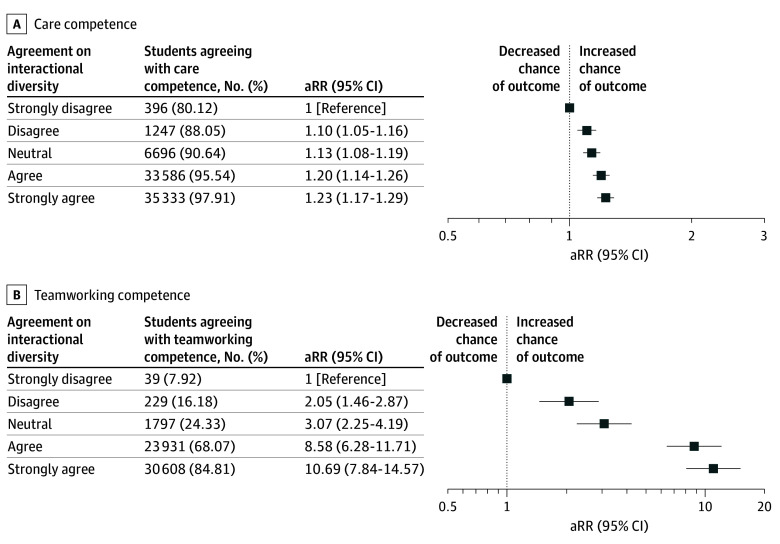
Care and Teamworking Competence Among White Medical Students by Meaningful Interactional Diversity Adjusted relative risks (aRRs) and 95% CIs for students’ self-reported competence to care for (A) and work with (B) people from different backgrounds, stratified by agreement with the meaningful interactional diversity item. Agreement reflects the extent to which students reported that their knowledge or opinions were influenced or changed by the perspectives of individuals from different backgrounds. Models adjust for sex, low-income status, sexual orientation, school ownership, region and matriculation year. The reference group is students who strongly disagreed.

Most students reported competence in caring for (77 258 [95.92%]) and working with (56 605 [70.28%]) people from different backgrounds. Higher levels of agreement on meaningful interactional diversity were associated with higher likelihood of both competencies. For care competence, aRRs increased from 1.13 (95% CI, 1.08-1.19) among those neutral to 1.23 (95% CI, 1.17-1.29) among those strongly agreeing, compared with those strongly disagreeing. The association with teamworking competence was particularly pronounced, with aRRs increasing from 3.07 (95% CI, 2.25-4.19) among those neutral to 10.69 (95% CI, 7.84-14.57) among those strongly agreeing, compared with those strongly disagreeing (Figure 3).

### Associations With Plans to Serve Underserved Communities

**Figure 4.**
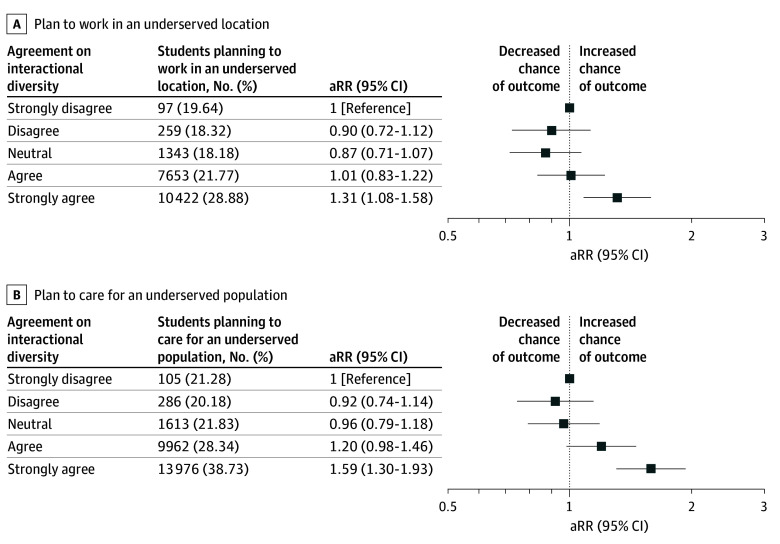
Intention to Serve Underserved Communities Among White Medical Students by Meaningful Interactional Diversity Adjusted relative risks (aRRs) and 95% CIs for intention to work in an underserved location (A) and care for an underserved population regardless of practice location (B), stratified by agreement with the meaningful interactional diversity item. Agreement reflects the extent to which students reported that their knowledge or opinions were influenced or changed by the perspectives of individuals from different backgrounds. Models adjust for sex, low-income status, sexual orientation, school ownership, region, and matriculation year. The reference group is students who strongly disagreed.

Overall, 19 775 students (24.55%) reported plans of working in underserved areas, and 25 941 (32.21%) reported plans of caring for underserved populations regardless of practice location. Compared with students who strongly disagreed with meaningful interactional diversity, only those who strongly agreed had higher adjusted risks of planning to work in underserved areas (aRR, 1.31; 95% CI, 1.08-1.58) and to care for underserved populations (aRR, 1.59; 95% CI, 1.30-1.93); estimates for students who disagreed, were neutral, or agreed did not differ from the reference group (Figure 4).

### Sensitivity Analyses and Adjacent-Level Contrasts

For all outcomes, both sensitivity analyses—one using complete cases and one restricted to the most recent two GQ cohorts—yielded results that were directionally consistent with the primary findings and showed similar estimate magnitudes. Adjacent-level contrasts across exposure categories showed a mostly stepwise pattern, with nonstatistically significant differences limited to lower-level comparisons for plans to serve underserved communities (eTable 6 in Supplement 1).

## Discussion

This study builds on the seminal work by Saha et al^5^ linking meaningful interactional diversity to White medical students’ intentions to practice in underserved areas and care competence across racial and ethnic backgrounds, using contemporary data and novel outcomes. Our findings suggest potential implications for the workforce readiness of White medical students—reflected in graded associations between meaningful interactional diversity and self-reported care and teamworking competence as well as personal and professional development—and, to a more limited extent, for population health, given that associations with intentions to work with underserved communities were observed only with a strong endorsement of meaningful interactional diversity. Together, these findings contribute to ongoing national efforts to understand how diversity may enhance medical training and preparedness to serve a diverse society.^26^

We observed novel associations between greater meaningful interactional diversity and improved self-reported clinical competencies among White medical students, namely care and teamworking competence (reflecting skills to care for and work with people from different backgrounds, respectively) and professional development (which the AAMC has deemed as a foundational competency to be instilled in medical school^19,20^). Diversity in medical school classrooms has been linked to a richer educational experience, including deeper discussions of medical conditions, broader perspectives offered during instruction, and greater exploration of alternative viewpoints.^44^ White medical school matriculants increasingly report that the diversity of a school’s student body and faculty is an important consideration in selecting a medical school.^45^ Institutions that foster meaningful interactions between students from different backgrounds are perceived as more supportive, positively influencing students’ sense of belonging and promoting their growth.^3,28,46,47^ Cross-cultural interaction may contribute to holistic growth and professional competence by fostering cognitive complexity, empathy, critical thinking, and leadership skills.^7,8,48^ However, there is evidence that the quality of these interactions matters greatly: positive interactions may be particularly beneficial for White students’ cognitive development and can offset the detrimental impact of negative interactions on cognitive development, which appear harmful to all students.^49^

Our findings suggest a link between meaningful cross-cultural engagement and personal development among White medical students. Given the rapidly diversifying US population^16^ and the reality of cultural differences between any 2 individuals,^34^ the ability to engage across differences and provide high-quality clinical care is essential for all physicians and impacts every patient and community. Cross-cultural engagement in good faith challenges stereotypic preconceptions,^44^ fostering personal growth, cultural humility, and competencies needed to mitigate bias and improve clinical decision-making,^33,34,50,51^ ultimately with the potential to better patient outcomes. Our findings support the theory that it may be the quality of engagement with diverse peers, rather than merely their presence, that fosters development and competence.^27,52^

Furthermore, our findings suggest that strong endorsement of meaningful cross-cultural interactions among White students—the largest racial subgroup in medical education—could potentially influence their motivation to serve underserved communities at graduation. However, these implications should be interpreted cautiously, as the predictive validity of these intention measures for eventual practice is unknown.^24^ Notably, only students most strongly endorsing meaningful interactional diversity exhibited statistically significant intentions to serve underserved communities, supporting prior theorization that a critical mass of diversity may be necessary to realize meaningful benefits.^5^

Taken together, these findings contribute to the literature bridging medical education with broader diversity science. It is plausible that the mechanisms identified in general higher-education settings by which meaningful cross-cultural interaction may confer benefit for majority groups (eg, perspective-sharing, stereotype and bias reduction, pluralistic reasoning, cognitive complexity, cultural knowledge)^7,11,53^ may also operate in the context of medical school.

### Limitations

This study has limitations. Meaningful interactional diversity was assessed based on White students’ agreement with a statement indicating that their knowledge or opinions had been influenced by individuals from different backgrounds. While this may suggest profound cross-cultural engagement, it does not directly capture the frequency of such interactions. A related and important limitation is the potential confounding by student attitudes or predispositions (eg, openness to diversity, social justice orientation, optimism, maturity, or social desirability). Given that both the exposure and outcomes are self-reported simultaneously, students with inherently positive or open-minded outlooks may have been more likely to report being influenced by diverse interactions and to rate their educational experiences positively. However, statistically significant associations were observed consistently across diverse outcome domains, and observed associations showed a graded, exposure-response association across distinct categories of meaningful interactional diversity, with the exception of service intention outcomes, for which associations were evident only at the strongest endorsement. Such incremental associations, rather than merely a contrast between extreme responders, strengthen the plausibility of a genuine pattern. Additionally, the meaningful interactional diversity item does not specify whether the influence was positive or negative, and this operationalization aligns with established approaches in higher education research.^5,27,28,29^ Similarly, the outcomes for personal and professional development reflect students’ perceptions of their institutions’ ability to nurture growth without accounting for individual efforts. While institutional responsibility is arguably central, students’ actual development may be higher or lower than perceived. Moreover, the teamworking competence measure reflects students’ perceptions that class diversity enhanced their training and skills to work with individuals from different backgrounds, rather than a direct assessment of teamworking ability. Furthermore, our reliance on self-reported data with variable levels of missingness introduces the potential for response bias. However, the study draws on the most comprehensive and widely used national dataset on allopathic medical students in the US and used multiple imputation to address missing data. Postimputation results closely aligned with complete case analyses but showed improved estimate stability. Despite the missing data, prioritizing students’ voices remains critical, particularly given their vulnerable position in the medical hierarchy.^54^ Additionally, this study did not adjust for confounders, such as the diversity climate of schools or states, which may shape the opportunities, frequency, and quality of cross-cultural engagement within the medical learning environment. Furthermore, as our sample includes students who matriculated only through 2022, findings may not reflect experiences shaped by more recent sociopolitical shifts. Additionally, although we observed demographic differences in levels of endorsement of meaningful interactional diversity, the reasons for these patterns were not examined in this study and remain unclear; their etiology and implications warrant future research. Additionally, the cross-sectional design limits causal inference.

## Conclusions

This cross-sectional study found that stronger endorsement of cross-cultural engagement that influenced or changed students’ knowledge or opinions (meaningful interactional diversity) was associated in a graded manner with White medical students’ personal and professional development as well as their competence to care for and work with people from different backgrounds, whereas associations with intentions to serve underserved communities were only evident among those reporting the strongest endorsement. Observing these associations among White students supports the premise that the educational benefits of diversity may extend beyond racially and ethnically minoritized groups. Future research should examine pathways and mechanisms underlying these associations and explore how to harness the benefits of diversity for all students, while continuing to center the well-being and success of historically marginalized students.

## Notes

Supplement 1. **eTable 1.** Variable missingness **eTable 2.** Raw frequencies of student and school characteristics by meaningful interactional diversity, including missingness **eTable 3.** Comparison of student and school characteristics in complete case vs imputed datasets **eTable 4.** Characteristics of White medical students and their schools by meaningful interactional diversity (complete case analysis) **eTable 5.** Association between school-level meaningful interactional diversity among White medical students and school-level structural diversity across race and ethnicity, income, and sexual orientation **eTable 6.** Adjusted relative risks for adjacent-level contrasts in meaningful interactional diversity across educational outcomes for White medical students

Supplement 2.
Data Sharing Statement
