# Supplementary material for: Meaningful Interactional Diversity, Professional Development, and Service Intent in White Medical Students
Source: JAMA Netw Open. 2026 Feb 20;9(2):e2560266. doi: 10.1001/jamanetworkopen.2025.60266 (PMC12924100; doi:10.1001/jamanetworkopen.2025.60266)
Supplement: Supplement 1. — eTable 1. Variable missingness eTable 2. Raw frequencies of student and school characteristics by meaningful interactional diversity, including missingness eTable 3. Comparison of student and school characteristics in complete case vs imputed datasets eTable 4. Characteristics of White medical students and their schools by meaningful interactional diversity (complete case analysis) eTable 5. Association between school-level meaningful interactional diversity among White medical students and school-level structural diversity across race and ethnicity, income, and sexual orientation eTable 6. Adjusted relative risks for adjacent-level contrasts in meaningful interactional diversity across educational outcomes for White medical students [file jamanetwopen-e2560266-s001.pdf]

Supplemental Online Content

Venkataraman S, Nguyen M, Hajduk AM, et al. Meaningful interactional diversity, professional development, and service intent in White medical students. *JAMA Netw Open.* 2026;9(2):e2560266. doi:10.1001/jamanetworkopen.2025.60266

eTable 1. Variable missingness

eTable 2. Raw frequencies of student and school characteristics by meaningful interactional diversity, including missingness

eTable 3. Comparison of student and school characteristics in complete case vs imputed datasets

eTable 4. Characteristics of White medical students and their schools by meaningful interactional diversity (complete case analysis)

eTable 5. Association between school-level meaningful interactional diversity among White medical students and school-level structural diversity across race and ethnicity, income, and sexual orientation

eTable 6. Adjusted relative risks for adjacent-level contrasts in meaningful interactional diversity across educational outcomes for White medical students

This supplemental material has been provided by the authors to give readers additional information about their work.

© 2026 Venkataraman S et al. *JAMA Network Open*.

eTable 1. Variable missingness

	Total number of participants	Participants with missing data, No. (%)
Low-income status	80 542	14 954 (18.57)
Sexual orientation	80 542	16 660 (20.68)
School region	80 542	923 (1.15)
School ownership	80 542	923 (1.15)
Meaningful interactional diversity	80 542	14 451 (17.94)
Personal development	80 542	15 935 (19.78)
Professional development	80 542	15 201 (18.87)
Care competence	80 542	14 159 (17.58)
Teamworking competence	80 542	14 462 (17.96)
Plan to work in an underserved location	80 542	15 847 (19.68)
Plan to serve an underserved population, regardless of practice location	80 542	15 842 (19.67)

© 2026 Venkataraman S et al. *JAMA Network Open*.

eTable 2. Raw frequencies of student and school characteristics by meaningful interactional diversity, including missingness

Characteristic	Participants by level of agreement with meaningful interactional diversity survey item, No. (%) (N=80 542)					
	**Strongly disagree, N=401**	**Disagree, N=1172**	**Neutral, N=5996**	**Agree, N=28 678**	**Strongly agree, N=29 844**	**Missing, N=14 451**
**Sex**						
Female	87 (0.23)	348 (0.90)	2215 (5.74)	14 060 (36.44)	16 209 (42.00)	5670 (14.69)
Male	314 (0.75)	824 (1.96)	3781 (9.01)	14 618 (34.84)	13 635 (32.50)	8781 (20.93)
**Low-income status**						
Yes	86 (0.48)	296 (1.64)	1412 (7.82)	6341 (35.10)	6383 (35.33)	3548 (19.64)
No	235 (0.49)	605 (1.27)	3197 (6.73)	16 882 (35.52)	18 625 (39.19)	7978 (16.79)
Missing	80 (0.53)	271 (1.81)	1387 (9.28)	5455 (36.48)	4836 (32.34)	2925 (19.56)
**Sexual orientation**						
Lesbian, gay, or bisexual	25 (0.41)	75 (1.22)	392 (6.36)	2397 (38.89)	3262 (52.92)	13 (0.21)
Heterosexual	340 (0.59)	1026 (1.78)	5254 (9.10)	25 252 (43.75)	25 740 (44.60)	106 (0.18)
Missing	36 (0.22)	71 (0.43)	350 (2.10)	1029 (6.18)	842 (5.05)	14 332 (86.03)
**School region**						
Central	106 (0.46)	382 (1.67)	1680 (7.34)	8530 (37.27)	7961 (34.79)	4227 (18.47)
Northeast	100 (0.47)	263 (1.24)	1542 (7.24)	7407 (34.78)	8480 (39.82)	3502 (16.45)
Southern	159 (0.57)	404 (1.46)	2102 (7.59)	9795 (35.39)	10 041 (36.28)	5176 (18.70)
Western	33 (0.43)	102 (1.31)	580 (7.47)	2654 (34.19)	3044 (39.22)	1349 (17.38)
Missing	3 (0.33)	21 (2.28)	92 (9.97)	292 (31.64)	318 (34.45)	197 (21.34)
**School ownership**						
Public	265 (0.51)	802 (1.53)	4112 (7.85)	19 134 (36.54)	18 483 (35.30)	9562 (18.26)
Private	133 (0.49)	349 (1.28)	1792 (6.57)	9252 (33.94)	11 043 (40.51)	4692 (17.21)
Missing	3 (0.33)	21 (2.28)	92 (9.97)	292 (31.64)	318 (34.45)	197 (21.34)

© 2026 Venkataraman S et al. *JAMA Network Open*.

eTable 3. Comparison of student and school characteristics in complete case vs. imputed datasets

Characteristic	Original (N= 50 884) Students, No. (%)	Imputed (N= 80 542) Students, No. (%)
**Sex**		
Female	26 094 (51.28)	38 589 (47.91)
Male	24 790 (48.72)	41 953 (52.09)
**Low-income**		
Yes	13 498 (26.53)	22 195 (27.56)
No	37 386 (73.47)	58 347 (72.44)
**Sexual orientation**		
Lesbian, gay, or bisexual	5103 (10.03)	7741 (9.61)
Heterosexual	45 781 (89.97)	72 801 (90.39)
**School region**		
Central	14 614 (28.72)	23 154 (28.75)
Northeast	14 021 (27.55)	21 549 (26.75)
Southern	17 104 (33.61)	27 981 (34.74)
Western	5145 (10.11)	7858 (9.76)
**School ownership**		
Public	33 100 (65.05)	52 955 (65.75)
Private	17 784 (34.95)	27 587 (34.25)
**Meaningful interactional diversity**		
Strongly Disagree	283 (0.56)	495 (0.61)
Disagree	814 (1.60)	1416 (1.76)
Neutral	4200 (8.25)	7387 (9.17)
Agree	21 831 (42.90)	35 155 (43.65)
Strongly Agree	23 756 (46.69)	36 089 (44.81)
**Personal development**		
Yes	37 167 (73.04)	58 483 (72.61)
No	13 717 (26.96)	22 059 (27.39)
**Professional development**		
Yes	47 487 (93.32)	74 791 (92.86)
No	3397 (6.68)	5751 (7.14)
**Care competence**		
Yes	48 876 (96.05)	77 258 (95.92)
No	2008 (3.95)	3284 (4.08)
**Teamworking competence**		
Yes	36 412 (71.56)	56 605 (70.28)
No	14 472 (28.44)	23 937 (29.72)
**Plan to work in an underserved location**		
Yes	12 792 (25.14)	19 775 (24.55)
No	38 092 (74.86)	60 767 (75.45)
**Plan to serve an underserved population, regardless of practice location)**		
Yes	16 851 (33.12)	25 941 (32.21)
No	34 033 (66.88)	54 601 (67.79)

© 2026 Venkataraman S et al. *JAMA Network Open*.

eTable 4. Characteristics of White medical students and their schools by meaningful interactional diversity (complete case analysis)

Characteristic	Participants by level of agreement with meaningful interactional diversity survey item, No. (%) (N=80 542)					
	**Strongly disagree, N=283**	**Disagree, N=814**	**Neutral, N=4200**	**Agree, N=21 831**	**Strongly agree, N=23 756**	**χ^2 test**
**Sex**						$\chi^2(4)=715.36$, $p<0.001$
Female	66 (0.25)	243 (0.93)	1589 (6.09)	11 017 (42.22)	13 179 (50.51)	
Male	217 (0.88)	571 (2.30)	2611 (10.53)	10 814 (43.62)	10 577 (42.67)	
**Low-income**						$\chi^2(4)=71.96$, $p<0.001$
Yes	77 (0.57)	272 (2.02)	1268 (9.39)	5890 (43.64)	5991 (44.38)	
No	206 (0.55)	542 (1.45)	2932 (7.84)	15 941 (42.64)	17 765 (47.52)	
**Sexual orientation**						$\chi^2(4)=117.83$, $p<0.001$
Lesbian, gay, or bisexual	23 (0.45)	55 (1.08)	319 (6.25)	1976 (38.72)	2730 (53.50)	
Heterosexual	260 (0.57)	759 (1.65)	3881 (8.48)	19 855 (43.37)	21 026 (45.93)	
**School region**						$\chi^2(12)=110.59$, $p<0.001$
Central	70 (0.48)	270 (1.85)	1192 (8.16)	6628 (45.35)	6454 (44.16)	
Northeast	70 (0.50)	187 (1.33)	1112 (7.93)	5746 (40.98)	6906 (49.26)	
Southern	120 (0.70)	277 (1.62)	1470 (8.60)	7357 (43.01)	7880 (46.07)	
Western	23 (0.45)	80 (1.55)	426 (8.28)	2100 (40.82)	2516 (48.90)	
**School ownership**						$\chi^2(4)=184.38$, $p<0.001$
Public	186 (0.56)	573 (1.73)	2925 (8.84)	14 666 (44.31)	14 750 (44.56)	
Private	97 (0.54)	241 (1.36)	1275 (7.17)	7165 (40.29)	9006 (50.64)	

© 2026 Venkataraman S et al. *JAMA Network Open*.

eTable 5. Association between school-level meaningful interactional diversity among White medical students and school-level structural diversity across race and ethnicity, income, and sexual orientation

School-level measures of structural diversity	School-level meaningful interactional diversity among White medical students, Mean % (SD) (N=155)		
	Yes, N=77	No, N=78	T-test
URiM	20.32 (9.37)	21.31 (22.55)	t(153)=0.36, p=0.72
Racial and ethnic minority	48.25 (12.09)	40.12 (21.15)	t(153)=-2.93, p=0.004
Low-income	28.89 (11.04)	32.19 (8.47)	t(153)=2.09, p=0.04
Lesbian, gay or bisexual	8.55 (4.10)	6.42 (2.05)	t(153)=-4.09, p<0.001

Note: This table is based on pre-imputation data. URiM and racial and ethnic minority status were defined using detailed self-reported race/ethnicity categories, consistent with AAMC policy classifications. These granular categories were not imputed due to small cell sizes and convergence issues. While race was imputed in other analyses using a harmonized numeric variable, URiM and racial and ethnic minority status could not be defined post-imputation using the original logic. Therefore, this table reflects the analytic sample pre-imputation. Yes versus no school-level meaningful interactional diversity was calculated by averaging student-reported meaningful interactional diversity within each school and splitting schools into two groups based on the median cutoff.

© 2026 Venkataraman S et al. *JAMA Network Open*.

eTable 6. Adjusted relative risks for adjacent-level contrasts in meaningful interactional diversity across educational outcomes for White medical students

	Educational outcomes, aRR (95% CI)					
**Adjacent-level contrasts**	**Personal development**	**Professional development**	**Care competence**	**Teamworking Competence**	**Plan to work in an underserved location**	**Plan to care for an underserved population, regardless of practice location**
Strongly agree (ref.) vs. Agree	0.83 (0.82-0.84)	0.95 (0.94-0.96)	0.97 (0.96-0.98)	0.80 (0.79-0.81)	0.77 (0.75-0.80)	0.75 (0.73-0.77)
Agree (ref.) vs. Neutral	0.72 (0.70-0.74)	0.87 (0.86-0.88)	0.95 (0.94-0.96)	0.36 (0.34-0.38)	0.87 (0.81-0.92)	0.81 (0.76-0.85)
Neutral (ref.) vs. Disagree	0.77 (0.72-0.83)	0.90 (0.87-0.94)	0.97 (0.95-0.99)	0.66 (0.58-0.77)	1.03 (0.91-1.18)	0.95 (0.85-1.07)
Disagree (ref.) vs. Strongly disagree	0.91 (0.78-1.06)	0.86 (0.79-0.94)	0.91 (0.86-0.96)	0.49 (0.35-0.68)	1.11 (0.89-1.39)	1.09 (0.88-1.35)

© 2026 Venkataraman S et al. *JAMA Network Open*.
