# Supplementary material for: Meaningful Interactional Diversity, Professional Development, and Service Intent in White Medical Students
Source: JAMA Netw Open. 2026 Feb 20;9(2):e2560266. doi: 10.1001/jamanetworkopen.2025.60266 (PMC12924100; doi:10.1001/jamanetworkopen.2025.60266)
Supplement: Supplement 2. — Data Sharing Statement [file jamanetwopen-e2560266-s002.pdf]

Data Sharing Statement

Venkataraman. Meaningful Interactional Diversity, Professional Development, and Service Intent in White Medical Students. *JAMA Netw Open*. Published February 20, 2026. doi:10.1001/jamanetworkopen.2025.60266

Data
Data available: No

Additional Information
Explanation for why data not available: The AAMC student questionnaire data analyzed in this study are available upon request through the AAMC's data request process (https://www.aamc.org/request-aamc-data).
